# Supplementary material for: How much do opinions regarding cultivated meat vary within the same country? The cases of São Paulo and Salvador, Brazil
Source: PLoS One. 2025 Feb 19;20(2):e0317956. doi: 10.1371/journal.pone.0317956 (PMC11838885; doi:10.1371/journal.pone.0317956)
Supplement: S1 Table — (DOCX) [file pone.0317956.s001.docx]

**S1 Table.** Odds ratios of providing a more positive response for each question, comparing variables gender, age, income, education and meat consumption.

| **Question** | **Variable** | **Odds ratio definition** | **Bivariate analysis** | **Multiple analysis** |
| --- | --- | --- | --- | --- |
| 9 | Gender | Men / Women | 1.34* | 1.23 |
|  | Age | 30 to 49 / 18 to 29 | 0.70* | 0.62* |
|  |  | 50 orolder / 18 to 29 | 0.72 | 0.60* |
|  | Income (R$) | 1997.00 to 4990.00 / Up to 1996.00 | 0.92 | 0.81 |
|  |  | Greater than 4990.00 / Upto 1996.00 | 1.52* | 1.25 |
|  | Education | University/ Elementary or High School | 1.39* | 1.33 |
|  |  | Postgraduate / Elementary or High School | 1.91* | 1.65 |
|  | Meat Consumption | Did not consume / 4 to 7 | 0.95 | 0.83 |
|  |  | 1 to 3 / 4 to 7 | 0.72* | 0.86 |
| 11 | Gender | Men / Women | 1.56* | 1.56* |
|  | Age | 30 to 49 / 18 to 29 | 0.72 | 0.54 |
|  |  | 50 or older / 18 to 29 | 0.37* | 0.23** |
|  | Income (R$) | 1997.00 to 4990.00 / Up to 1996.00 | 1.05 | 0.81 |
|  |  | Greater than 4990.00 / Up to 1996.00 | 1.78* | 1.46 |
|  | Education | University/ Elementary or High School | 1.30 | 1.24 |
|  |  | Postgraduate / Elementary or High School | 1.36 | 1.00 |
|  | Meat Consumption | Did not consume / 4 to 7 | 0.07* | 0.04** |
|  |  | 1 to 3 / 4 to 7 | 0.53* | 0.67 |
| 12 | Gender | Men / Women | 1.35* | 1.35* |
|  | Age | 30 to 49 / 18 to 29 | 1.28 | 1.24 |
|  |  | 50 or older / 18 to 29 | 1.17 | 1.19 |
|  | Income (R$) | 1997.00 to 4990.00 / Up to 1996.00 | .1.08 | 1.12 |
|  |  | Greater than 4990.00 / Up to 1996.00 | 0.85 | 0.91 |
|  | Education | University/ Elementary or High School | 0.93 | 0.99 |
|  |  | Postgraduate / Elementary or High School | 0.97 | 1.07 |
|  | Meat Consumption | Did not consume / 4 to 7 | 1.17* | 0.17* |
|  |  | 1 to 3 / 4 to 7 | 1.21 | 1.19 |
| 13 | Gender | Men / Women | 1.10 | 1.06 |
|  | Age | 30 to 49 / 18 to 29 | 0.81 | 0.75 |
|  |  | 50 or older / 18 to 29 | 0.56* | 0.45* |
|  | Income (R$) | 1997.00 to 4990.00 / Up to 1996.00 | 1.51* | 1.42* |
|  |  | Greater than 4990.00 / Up to 1996.00 | 1.87* | 1.76* |
|  | Education | University/ Elementary or High School | 1.26 | 1.00 |
|  |  | Postgraduate / Elementary or High School | 1.44 | 1.04 |
|  | Meat Consumption | Did not consume / 4 to 7 | 0.78 | 0.76 |
|  |  | 1 to 3 / 4 to 7 | 0.67* | 0.83 |
| 14 | Gender | Men / Women | 1.62* | 1.48* |
|  | Age | 30 to 49 / 18 to 29 | 1.47* | 1.46* |
|  |  | 50 or older / 18 to 29 | 1.37 | 1.45 |
|  | Income (R$) | 1997.00 to 4990.00 / Up to 1996.00 | 0.92 | 0.85 |
|  |  | Greater than 4990.00 / Up to 1996.00 | 1.34 | 1.11 |
|  | Education | University/ Elementary or High School | 1.17 | 1.16 |
|  |  | Postgraduate / Elementary or High School | 0.93 | 0.79 |
|  | Meat Consumption | Did not consume / 4 to 7 | 0.20** | 0.20** |
|  |  | 1 to 3 / 4 to 7 | 0.66* | 0.67* |
| 15 | Gender | Men / Women | 1.03 | 0.96 |
|  | Age | 30 to 49 / 18 to 29 | 0.86 | 0.87 |
|  |  | 50 or older / 18 to 29 | 0.70 | 0.68 |
|  | Income (R$) | 1997.00 to 4990.00 / Up to 1996.00 | 1.28 | 1.15 |
|  |  | Greater than 4990.00 / Up to 1996.00 | 1.45 | 1.32 |
|  | Education | University/ Elementary or High School | 1.30 | 1.14 |
|  |  | Postgraduate / Elementary or High School | 0.85 | 0.71 |
|  | Meat Consumption | Did not consume / 4 to 7 | 0.50 | 0.52 |
|  |  | 1 to 3 / 4 to 7 | 0.66* | 0.75 |
| 16 | Gender | Men / Women | 1.52* | 1.55* |
|  | Age | 30 to 49 / 18 to 29 | 1.26 | 1.30 |
|  |  | 50 or older / 18 to 29 | 1.39 | 1.58* |
|  | Income (R$) | 1997.00 to 4990.00 / Up to 1996.00 | 0.75 | 0.79 |
|  |  | Greater than 4990.00 / Up to 1996.00 | 0.64* | 0.68 |
|  | Education | University/ Elementary or High School | 0.84 | 0.98 |
|  |  | Postgraduate / Elementary or High School | 0.61* | 0.73 |
|  | Meat Consumption | Did not consume / 4 to 7 | 0.14* | 0.14* |
|  |  | 1 to 3 / 4 to 7 | 1.18 | 1.07 |
| 17 | Gender | Men / Women | 1.14 | 1.02 |
|  | Age | 30 to 49 / 18 to 29 | 0.92 | 0.84 |
|  |  | 50 or older / 18 to 29 | 0.55* | 0.46* |
|  | Income (R$) | 1997.00 to 4990.00 / Up to 1996.00 | 1.55* | 1.37 |
|  |  | Greater than 4990.00 / Up to 1996.00 | 2.19** | 1.88* |
|  | Education | University/ Elementary or High School | 1.39* | 1.15 |
|  |  | Postgraduate / Elementary or High School | 1.81* | 1.26 |
|  | Meat Consumption | Did not consume / 4 to 7 | 0.29* | 0.29* |
|  |  | 1 to 3 / 4 to 7 | 0.56** | 0.71* |
