# Supplementary material for: How much do opinions regarding cultivated meat vary within the same country? The cases of São Paulo and Salvador, Brazil
Source: PLoS One. 2025 Feb 19;20(2):e0317956. doi: 10.1371/journal.pone.0317956 (PMC11838885; doi:10.1371/journal.pone.0317956)
Supplement: Attachment — Submitted filename: Response to Reviewers.docx [file pone.0317956.s003.docx]

Curitiba, November 18^th^, 2024

Dear Editor,

We sincerely appreciate your careful consideration of our manuscript and the constructive feedback provided by the reviewers. We are pleased to know that the work has merit, and we thank you for the invitation to submit a revised version addressing the points raised during the review process.

We have carefully considered all recommendations and have addressed each one. We believe these revisions result in a significantly improved manuscript.

We confirm that no changes were made to our financial disclosure statement, and we followed the guidelines for resubmitting the figure files.

We have addressed all requirements for the revised submission:

1. Style requirements: Our manuscript now meets PLOS ONE's formatting guidelines, based on the templates provided.
2. Participant consent: We added details regarding the type of consent obtained (lines 148-151).
3. Data availability statement: Our data availability statement reflects our data sharing plan, in compliance with PLOS ONE’s policy. Data is available at the link: <https://figshare.com/articles/dataset/Full_dataset_with_the_participation_of_respondents_from_S_o_Paulo_and_Salvador_from_September_5th_to_23rd_2019_/27105952?file=49425988>, mentioned in "supporting information" (lines 760-765).
4. Ethics statement: We have ensured that the ethics statement appears only in the Methods section, as per the journal’s guidelines (lines 144-146).

Dear Reviewers,

We would like to express our sincere gratitude for your thorough evaluation and the detailed feedback provided. We deeply appreciate the valuable suggestions that have helped improve the quality and clarity of the manuscript.

We have carefully considered and addressed each recommendation. We are confident that the revisions have resulted in a significantly enhanced manuscript.

To facilitate understanding, we have highlighted the reviewers' comments in red and our responses in black.

Reviewer 1:

Abstract

The Abstract Section provides a clear objective and concrete data to compare consumer intentions regarding cultivated meat in São Paulo and Salvador, Brazil. However, it lacks sufficient detail about the methodology, such as the type of questions used in the survey and how they measure consumer intent. The analysis of the results is superficial, offering little insight into why economic factors might influence awareness and acceptance of cultivated meat. Additionally, the conclusion is overly generic, stating that cultivated meat can solve problems related to conventional meat without specifying how. The structure could also be improved for better coherence, especially when explaining regional differences.

Regarding the methodological details mentioned in the abstract, we included the following information: “An online questionnaire with 17 multiple-choice and open questions regarding opinions on conventional and cultivated meat was used and the results were analyzed both quantitatively and qualitatively” (lines 31-33).

To address the superficiality of the results and the generic conclusion presented in the abstract, we added: “Such disparity in awareness seems coherent with differences in access to information and educational levels. Our results suggest that the acceptance of cultivated meat varies significantly across different regions of Brazil, likely related to the country’s continental size, uneven economic and educational status and rich cultural diversity. We conclude that the acceptance of cultivated meat correlates with knowledge about it and that efforts to raise such knowledge require the consideration of cultural and socioeconomic aspects on a regional rather than national level, especially for geographically big and culturally diverse countries. Future research seems warranted as additional reasons for different acceptance levels likely play key roles and may be investigated in target groups less diverse in educational levels. Continued research is also essential due to dynamics of acceptance and its entanglement with familiarity and knowledge regarding cultivated meat, both of which tend to significantly increase with time” (lines 37-48).

Introduction

The Introduction Section effectively presents the growing global demand for meat, highlighting key environmental, ethical, and health concerns linked to conventional animal production. It emphasizes the importance of consumer acceptance for cultivated meat's success, particularly in Brazil's context. The global relevance of the topic and the framing of cultivated meat as a potential solution to these issues provide a solid foundation for the study. Despite that, some topic shifts (e.g., from environmental issues to cellular agriculture) are abrupt. More natural transitions would improve text cohesion.

To improve the flow of the text and ensure a more natural transition between ideas, we restructured the paragraphs, adding linking phrases to strengthen textual cohesion. This reformulation aims to facilitate reading and comprehension, promoting a smoother progression of arguments and improving continuity between the topics discussed. By inserting these connections, we seek to ensure a consistent narrative that enhances clarity and logical linkage between the paragraphs.

Consumer resistance to alternative proteins is mentioned but not fully explored. It would be useful to provide more detail, such as risk perception or cultural barriers, with concrete examples.

We further explore consumer resistance to alternative proteins by providing additional details and references in: “It is known that price is a major driving force for consumer buying decisions [20]. Would then price-parity between alternative proteins and conventional animal products suffice for a major market shift towards alternative proteins? Maybe not. Meat consumers are reported to be resistant to alternative proteins due to a variety of reasons. An important reason seems to be their perception of risks in novel foods [18]. For instance, consumer preferences and risk perceptions influence the intention to try alternative proteins, and the greater the risk perceived the less likely consumers are to try them [21]. In addition, consumers who are skeptical when it comes to the decision of consuming cultivated meat report feelings of disgust, lack of naturalness, and negative sensory expectations [22, 23, 24, 25, 26, 27]. Food neophobia, defined as an individual's reluctance to consume new foods [18], is a general reason for rejecting any food item on the basis of its novelty and unfamiliarity which also plays a role for rejecting alternative proteins” (lines 100-111).

Since there is no a Theoretical Background Section, I would have expected more references to existing literature to support the aims of the manuscript.

To improve the Theoretical Background, we added more studies as references throughout the Introduction section: “In this sense, the problems involved in the current scenario of animal production indicate a need to adapt to more sustainable systems that also value aspects related to animal welfare [5], with concerns about such topics growing among consumers [6]. Coherently, concerns about the ethics and environmental impacts of traditional meat consumption have stimulated the development of alternatives [5], and protein source diversification emerges as a promising option, with the potential to generate environmental benefits [7] and higher standards regarding animal suffering [6]. Furthermore, alternative proteins address issues related to public health, food safety, and animal welfare [5, 6, 8, 9, 10]. However, there are challenges for their development. For instance, although cultivated meat uses significantly less land and water than conventional production, it may require higher energy inputs, depending on which specific chains are compared [11]. The climate effects of cultivated meat can also vary depending on the energy source used [8]. Biotechnological challenges for scaling up cultivated meat production in a cost-effective way remain [12], while the use of animal-free inputs presents its own set of research questions [13]. Yet another example is the need to understand consumer demands and intention to purchase cultivated meat products [14], as an evident essential part for the success of the new food production systems. Considering the remaining difficulties, the robust and multidimensional perceived benefits of cultivated meat and other alternative proteins boost increasing private and public investments for the resolution of the remaining challenges” (lines 66-84); “As a response to the need for science-based solutions, a new field of research, education, outreach activities and production systems conceived to develop and deliver alternative proteins, commonly denominated cellular agriculture, is currently under development [15]. In turn, alternative proteins are foods produced through the cultivation of animal cells *ex vivo* (i.e. out of the body), fermentation processes such as biomass and precision fermentation, or a combination of plant ingredients, with the goal of offering animal products or their analogues without the need for animal farming and slaughter [16]. Cultivated meat is a product of the culture of muscle tissue from a collection of cells from a live animal [17]” (lines 85-92); “This price-parity seems particularly difficult to achieve unless fundamental shifts in subsidies currently available for conventional meat are enacted [19]. It is known that price is a major driving force for consumer buying decisions [20]” (lines 98-100); “In addition, consumers who are skeptical when it comes to the decision of consuming cultivated meat report feelings of disgust, lack of naturalness, and negative sensory expectations [22, 23, 24, 25, 26, 27]. Food neophobia, defined as an individual's reluctance to consume new foods [18], is a general reason for rejecting any food item on the basis of its novelty and unfamiliarity which also plays a role for rejecting alternative proteins” (lines 106-111); “It is perceived that consumer acceptance will dictate its success [6, 30], in addition to being a determinant of the new balance between conventional and cultivated meat production chains. In this sense, whether cellular agriculture will become more than just a niche market [31] depends critically on the percentage of consumers that buy its products” (lines 119-123); “It is logical, then, that cultivated meat purchase intention be a relevant research topic. Indeed, the intention to consume cultivated meat has been the focus of scientific papers within the last decade, with first publications appearing in 2015 [32]. The levels of acceptance of cultivated meat vary among different research results worldwide [22, 33, 34, 35], as in Brazil [31, 36, 37, 38]” (lines 124-128).

The hypothesis about "significant differences" in the intention to consume cultivated meat across regions is somewhat vague. It would be helpful to specify potential influencing variables, such as food culture, economic status, or media exposure.

To better explain the differences in the intention to consume cultivated meat across regions, we adapted the following paragraph: “Brazil hosts around half the South American population and around half its geographical area. In addition, Brazil is the largest exporter of beef and the second largest producer of meat in the world [39], being arguably considered a country with a barbecue culture [36]. As agriculture and animal production constitute a major component of the country’s Gross Domestic Product (GDP), the introduction of meat alternatives nationally, e.g. cultivated meat, seems essential to maintain its market share in the future, requiring careful planning to maximize the benefits and mitigate the disadvantages [40]. Thus, a more detailed regional understanding of Brazilians’ perceptions of and intention to consume alternative proteins, with their underlying reasons, seems both relevant and a rich study case scenario. Brazilian regions differ significantly in geographic, socioeconomic, food culture, and media exposure aspects and it is likely that such differences significantly influence consumer acceptance of new foods. Indeed, in a previous study comparing two Brazilian cities within the same geographical region of Brazil, the South, significant differences in consumption intention were observed [31], raising interesting scientific questions regarding the magnitude of differences which may appear when the comparison is expanded to include cities from other regions in the country” (lines 132-146) and “Salvador, the largest urban center in the Northeast region and the capital of the State of Bahia, with a population of 2,417,678 inhabitants [41], is currently ranked with the weakest economic and employment indicators among Brazilian State capitals [42]. In contrast, the city of São Paulo, capital of the Southeastern State of São Paulo, is the most populous city in Brazil, with a population of 11,451,99 inhabitants [41], and stands out as the most economically developed center in the country [42]” (lines 157-162).

To enhance the readability of the text, it would be beneficial to include an overview of the manuscript’s structure at the end of the Introduction Section. This would help guide readers through the content and provide a clearer understanding of how the study will unfold, making it easier to follow the main arguments and findings presented later in the paper.

We summarized the structure of the manuscript at the end of the introduction: “This paper is structured to provide a demographic characterization of our participants, followed by descriptive and qualitative data obtained from their responses, which were later summarized and prepared for comparative and explanatory analyses, according to appropriate methodology for each case. Results were then statistically compared between cities and also analyzed considering relevant demographic data as potential explanatory factors. There follows a discussion section, organized from general to more specific issues, which includes the limitations of our study and potential priorities for further research in the field” (lines 163-170).

Material and Methods

The materials and methods section presents a clear and well-structured methodology, detailing the study's design, participant recruitment, and survey administration, which helps readers understand the research process. It emphasizes ethical considerations by including information about ethical approval and participant consent, adding credibility to the study. The comprehensive survey design, incorporating both multiple-choice and open-ended questions, allows for a nuanced understanding of consumer attitudes toward cultivated meat and facilitates the collection of both quantitative and qualitative data. Additionally, the robust statistical analysis methods used, such as binary logistic regression and ordinal regression, indicate a thorough approach to data analysis, enhancing the reliability of the findings. Although a list of questions is provided, a brief explanation of how specific questions relate to the study's objectives could help readers understand their significance.

An explanation of how each questionnaire question relates to the study's objective was incorporated in: “In addition to questions specifically about cultivated meat acceptance, we selected related issues that may provide insights into respondents' acceptance levels. The degree of familiarity with cultivated meat is likely a key factor in understanding consumer interest. Similarly, capturing respondents' perceptions of the positive and negative impacts of conventional meat on environmental, human health, and animal welfare issues may help interpreting their motivations for accepting or rejecting meat alternatives. Thereby, questions 8 to 17 (Table 1) explore participants' level of knowledge about conventional and cultivated meat, their intention to consume cultivated meat, and their perceptions of how the production of either one affects the environment, human health, and animal welfare” (lines 186-195).

There is no discussion of potential biases in the sampling or response processes. Addressing limitations related to participant selection or self-reported data would provide a more balanced view of the findings.

The limitations and potential biases of the respondents were included in the discussion session of the article: “Some potential biases and limitations may have influenced the results of this work. The sampling, while extensive in two large Brazilian cities, may not fully represent the general population. The online questionnaire may have attracted more participants with internet access and familiarity with or at least curiosity about cultivated meat. In addition, the reliance on limited respondent stratification may relate to subgroups of the population not being represented in our survey, which requires caution in making inferences for the specific cities studied and even more so for the country in general. The limited number of questions in the survey, especially open-ended questions, is also a limitation, which combined with the need for extracting key words may result in an oversimplification of respondent views. The self-reported data may exhibit social desirability bias, where respondents provide answers that they consider socially acceptable, susceptible to personal interpretations and selective memory [74, 75]. Additionally, the lack of prior knowledge about cultivated meat intrinsically limited the strength of rationales supporting the responses regarding the perceptions of its potential impacts regarding the environment, human health, and animal welfare. An additional intrinsic issue relates to the dynamic character of the acceptance of cultivated meat, even more marked as our results show the significance of knowledge and familiarity, both of which tend to increase with time. This means that the picture our data set shows is best understood as historical than as descriptive of today’s scenario. These limitations underscore the complexities of gathering data and interpreting public opinion on cultivated meat, also exposing the need for continued research” (lines 469-488).

To enhance clarity and improve the presentation of information, I would suggest adding a table to summarize the explanatory variables considered in the study. This table could neatly categorize the variables such as city (São Paulo, Salvador), gender (male, female), age groups (18 to 29, 30 to 49, 50 or older), income brackets (up to R$1996.00, R$1997.00 to R$4990.00, more than R$4990.00), education levels (elementary/high school, higher education, postgraduate degree), and frequency of meat consumption (does not eat, 1 to 3 days a week, 4 to 7 days a week). A visual representation of these factors would make it easier for readers to grasp the demographic breakdown and the variables being analyzed in the study.

To avoid data duplication, we refer to Table 2, where the demographic variable categories are already presented (line 250-252).

Results

The Results Section includes a comprehensive presentation of demographic data, clear statistical reporting with specific percentages, and effective cross-comparison between cities, highlighting regional differences in awareness and attitudes toward cultivated meat. Additionally, the reference to figures enhances visual comprehension of the data.

The Authors might consider adding tables to present the descriptive statistics for socio-demographic and behavioral variables. This approach would not only make the data more accessible and easier to understand but also allow you to reduce the text by directing readers to the tables for more detailed information. This way, the text would be more concise and focused on the main results, improving the overall readability of the section.

According to the suggestion, we included tables to present the results of the descriptive statistics, making the data more accessible and easier to understand. The following tables were added: “Table 2. Demographic results by response categories of the 809 respondents from an online questionnaire administered from September 5 to 23, 2019, to residents of São Paulo and Salvador, Brazil” (lines 250-252); “Table 3. Answers to the question “Have you heard about cultivated meat?”, given by 809 respondents from São Paulo and Salvador, Brazil, in an online questionnaire from September 5 to 23, 2019” (lines 263-265); “Table 4. Answers to the question “Would you eat cultivated meat”, given by 809 respondents from São Paulo and Salvador, Brazil, in an online questionnaire from September 5 to 23, 2019” (lines 272-274); “Table 5. Responses to questions about the relationship between cultivated meat and the environment, human health, and animal welfare, based on respondents' familiarity of cultivated meat, collected from 809 participants in São Paulo and Salvador, Brazil, in an online questionnaire conducted from September 5 to 23, 2019” (lines 296-299).

Discussion

The Discussion Section effectively places the study's results in the broader context of Brazil’s socio-economic landscape, comparing São Paulo and Salvador, which helps explain the differences in awareness and acceptance of cultivated meat. The author provides a valuable comparison of their findings with those from studies conducted in other countries, highlighting significant disparities in acceptance rates and contextualizing these within the socio-cultural frameworks of the regions.

Despite that, in my opinion the Discussion Section is quite lengthy and could be more concise. Streamlining certain sections would improve readability and focus on the main findings without diluting the content. Furthermore, such section should adopt a more critical approach to interpreting the findings.

To make the discussion more concise, we streamlined certain sections to improve readability and emphasize the results. Moreover, we adopted a more critical approach, making targeted adjustments to enhance clarity and depth. However, the net result of reviewers’ requests is the inclusion of additional paragraphs in the discussion section, which unfortunately conflicted with trying to reduce its size.

While the discussion refers to previous studies, providing more specific data points or direct comparisons in terms of percentages or numbers would strengthen the argument and support the claims being made.

We incorporated numerical and percentage data from previous studies to strengthen the discussion: “where 16.0% of the population was unemployed in 2019” (lines 336-337); “with a score of 8.67” (line 342); “with a score of 6.05” (line 343); “A study conducted in Germany with 713 participants showed that 38.0% of them already had prior knowledge of cultivated meat and demonstrated greater acceptance [33]. A study with 1,296 participants in the Netherlands indicated that 79.0% had never heard of cultivated meat. However, after explaining the technique and its possible advantages and disadvantages, 52.0% claimed to be willing to try it [47]. Additionally, a study conducted with 739 participants in the southern region of Brazil showed that 39.3% of them responded positively to the questions after watching an explanatory video about cellular agriculture [31]” (lines 345-352); “A study conducted with 214 participants in Canada, where 38% believed that cultivated meat would not have the same flavor as conventional meat, while 30% stated that it would have the same taste [48]” (lines 375-377); “Compared to conventional meat production, cultivated meat is associated with approximately 7–45% less energy, 78–96% lower greenhouse gas emissions, 99% lower land use, and 82–96% lower water use, depending on the product compared [11, 21, 61]” (lines 412-415).

Conclusion

The Conclusion Section could benefit from a more critical engagement with the implications of the findings. For example, discussing the challenges of disseminating knowledge about cultivated meat and the potential barriers to acceptance could provide a more balanced view.

To address the suggestions for greater critical engagement with the implications of the results, we restructured the conclusion section. We discussed the challenges in knowledge dissemination and the potential barriers to the acceptance of cultured meat: “The acceptance of cultivated meat varies significantly across Brazil, likely due to the country’s continental size, economic disparity, and rich cultural diversity. This variation suggests that the dissemination of knowledge about cultivated meat may encounter barriers related to regional public understanding, accessibility, and preferences. Our results have direct implications, as they make such challenges evident, and expose the need to considerate regional identities when promoting cultivated meat, as resistance may stem from unfamiliarity or concerns about changes to food traditions. It remains to be studied whether variations emerge in countries with different geographical, demographic and cultural profiles” (lines 491-498).

While the conclusion touches on the need for regional considerations, it could elaborate on how these findings might impact policy, industry practices, and consumer education initiatives.

We introduced the following paragraph: “Our findings highlight the importance of regional considerations not only for academic research but also for practical applications in public policy, industry practices, and consumer education initiatives. The efficiency of public policies will benefit from targeted educational initiatives that address the specific concerns of different regions, while industry practitioners might adjust their outreach strategies to be more culturally sensitive. In addition, collaborating with local institutions is likely to foster a more productive approach to introducing cultivated meat” (lines 499-505).

A brief mention of the study's limitations could strengthen the Conclusion by providing transparency and acknowledging the complexities of interpreting the results.

We have included a comprehensive paragraph on the limitations of our study, as this is an important suggestion for the improvement of our paper. In order to keep clear and objective conclusion, we opted to include the limitations at the end of the discussion section.

While the call for further research is commendable, offering specific areas or questions for future research could enhance the utility of the conclusion for researchers looking to build on this work.

We introduced the following paragraph: “Future studies are warranted to explore the influence of different demographic factors on acceptance, assess the efficacy of targeted educational strategies in enlightening perceptions, and track opinion shifts over time as knowledge and availability of cultivated meat increase. Such research will be invaluable in fostering public understanding, addressing misconceptions, supporting public policies and facilitating informed decision-making regarding cultivated meat and other alternative proteins” (lines 506-511).

Reviewer 1, we deeply appreciate the thorough evaluation conducted, which significantly contributed to the improvement of the work. We considered all the suggestions presented and implemented the necessary adjustments to strengthen the analysis and clarity of the results. Furthermore, we express our sincere gratitude for the compliments mentioned in each section, which not only acknowledge the effort invested but also reinforce the quality of the research. Each comment was carefully reflected upon and incorporated to ensure that the work met the highest standards of excellence set by PLoS One.

Reviewer 2:

I recommend reconsidering the article's title in light of the question: Do opinions regarding cultivated meat vary within the same country? It seems self-evident that opinions differ among individuals, as these are shaped by a variety of factors. Even opinions within members of the same family can differ, so it is expected that opinions across a country, especially one as diverse as Brazil, would vary significantly due to its vast population and territory. Additionally, given Brazil's ex-tensive geographical area, putting “the case of Brazil” while the study to only two cities may not be advisable, as these cities may not be representative of the broader population. It may be useful to reflect this in the title, indicating that the study pertains to specific cities in Brazil.

We revised the title of the work, following the suggestion provided, and changed it to: “How much do opinions regarding cultivated meat vary within the same country? The cases of Salvador and São Paulo, Brazil” (lines 4-5).

In the abstract and introduction, while the authors summarize the main research question and key findings, it would be beneficial to include more details about the research methodology used.

Regarding the methodological details suggested for the abstract, we have added the following information: “An online questionnaire with 17 multiple-choice and open questions regarding opinions on conventional and cultivated meat was used and the results were analyzed both quantitatively and qualitatively” (lines 31-33).

Furthermore, while the authors mention that there is extensive literature on the subject, they do not clearly explain how this study relates to or builds upon previously published research.

To enhance the theoretical background, we have included additional works as references throughout the Introduction section: “In this sense, the problems involved in the current scenario of animal production indicate a need to adapt to more sustainable systems that also value aspects related to animal welfare [5], with concerns about such topics growing among consumers [6]. Coherently, concerns about the ethics and environmental impacts of traditional meat consumption have stimulated the development of alternatives [5], and protein source diversification emerges as a promising option, with the potential to generate environmental benefits [7] and higher standards regarding animal suffering [6]. Furthermore, alternative proteins address issues related to public health, food safety, and animal welfare [5, 6, 8, 9, 10]. However, there are challenges for their development. For instance, although cultivated meat uses significantly less land and water than conventional production, it may require higher energy inputs, depending on which specific chains are compared [11]. The climate effects of cultivated meat can also vary depending on the energy source used [8]. Biotechnological challenges for scaling up cultivated meat production in a cost-effective way remain [12], while the use of animal-free inputs presents its own set of research questions [13]. Yet another example is the need to understand consumer demands and intention to purchase cultivated meat products [14], as an evident essential part for the success of the new food production systems. Considering the remaining difficulties, the robust and multidimensional perceived benefits of cultivated meat and other alternative proteins boost increasing private and public investments for the resolution of the remaining challenges” (lines 66-84); “As a response to the need for science-based solutions, a new field of research, education, outreach activities and production systems conceived to develop and deliver alternative proteins, commonly denominated cellular agriculture, is currently under development [15]. In turn, alternative proteins are foods produced through the cultivation of animal cells *ex vivo* (i.e. out of the body), fermentation processes such as biomass and precision fermentation, or a combination of plant ingredients, with the goal of offering animal products or their analogues without the need for animal farming and slaughter [16]. Cultivated meat is a product of the culture of muscle tissue from a collection of cells from a live animal [17]” (lines 85-92); “This price-parity seems particularly difficult to achieve unless fundamental shifts in subsidies currently available for conventional meat are enacted [19]. It is known that price is a major driving force for consumer buying decisions [20]” (lines 98-100); “In addition, consumers who are skeptical when it comes to the decision of consuming cultivated meat report feelings of disgust, lack of naturalness, and negative sensory expectations [22, 23, 24, 25, 26, 27]. Food neophobia, defined as an individual's reluctance to consume new foods [18], is a general reason for rejecting any food item on the basis of its novelty and unfamiliarity which also plays a role for rejecting alternative proteins” (lines 106-111); “It is perceived that consumer acceptance will dictate its success [6, 30], in addition to being a determinant of the new balance between conventional and cultivated meat production chains. In this sense, whether cellular agriculture will become more than just a niche market [31] depends critically on the percentage of consumers that buy its products” (lines 119-123); “It is logical, then, that cultivated meat purchase intention be a relevant research topic. Indeed, the intention to consume cultivated meat has been the focus of scientific papers within the last decade, with first publications appearing in 2015 [32]. The levels of acceptance of cultivated meat vary among different research results worldwide [22, 33, 34, 35], as in Brazil [31, 36, 37, 38]” (lines 124-128).

Regarding the results, I recommend presenting the results in tabular form for clarity and ease of interpretation.

Following the suggestion, we have included tables to present the results, making the data clearer and easier to interpret. The following tables were added: “Table 2. Demographic results by response categories of the 809 respondents from an online questionnaire administered from September 5 to 23, 2019, to residents of São Paulo and Salvador, Brazil” (lines 250-252); “Table 3. Answers to the question “Have you heard about cultivated meat?”, given by 809 respondents from São Paulo and Salvador, Brazil, in an online questionnaire from September 5 to 23, 2019” (lines 263-265); “Table 4. Answers to the question “Would you eat cultivated meat”, given by 809 respondents from São Paulo and Salvador, Brazil, in an online questionnaire from September 5 to 23, 2019” (lines 272-274); “Table 5. Responses to questions about the relationship between cultivated meat and the environment, human health, and animal welfare, based on respondents' familiarity of cultivated meat, collected from 809 participants in São Paulo and Salvador, Brazil, in an online questionnaire conducted from September 5 to 23, 2019” (lines 296-299).

For questions 10, 11, 15, and 17, if respondents have not previously heard about cultivated meat, may provide inconsistent responses due to their lack of knowledge on the subject, making the data less reliable for statistical analysis and reducing the comparability between respondents’ answers due to varied interpretations of the terms. Therefore, comparing responses from individuals familiar with cultivated meat to those from individuals unfamiliar with it may not be advisable. I suggest expanding the descriptive analysis to include segmentation, for example, those who consume meat versus those who do not, and those who are familiar with cultivated meat versus those who are not. Presenting a cross-tabulation that includes sociodemographic variables would also help to better understand the differences between these groups.

We expanded the descriptive analysis by comparing the responses to questions 10, 11, 13, 15, and 17, conducting a comparison between individuals with and without knowledge of cultured meat, and analyzing their opinions on the environment, human health, and animal welfare in relation to cultured meat. Additionally, we included a table with the results obtained.

“Among the 310 respondents who had heard of cultivated meat, 156 (50.3%) said they would eat it, 64 (20.7%) said they would not, and 90 (29.0%) said they didn’t know. Of the 499 respondents who were unfamiliar with cultivated meat, 109 (21.8%) said they would eat it, 147 (29.5%) said they would not, and 243 (48.7%) said they didn’t know (Table 5)” (lines 291-294).

“Table 5. Responses to questions about the relationship between cultivated meat and the environment, human health, and animal welfare, based on respondents' familiarity of cultivated meat, collected from 809 participants in São Paulo and Salvador, Brazil, in an online questionnaire conducted from September 5 to 23, 2019” (lines 296-299).

Furthermore, it is important to provide detailed information regarding the steps taken to generate the word clouds, particularly the criteria used in the process. Care must be taken to avoid excessive reductionism, ensuring that the core meaning of the responses is retained. While using a thesaurus can be helpful in word cloud construction, its application must be handled carefully to avoid distorting the data. Additionally, it could be beneficial to conduct an additional analysis by excluding certain custom words and exploring new, potentially significant terms that may emerge in the word clouds.

We confirmed that the terms selected for the word cloud are aligned with a thesaurus. Additionally, we provide more details on how the word cloud was constructed: “The answers to the open-ended questions were analyzed by two researchers, who initially synthesized the phrases individually into preliminary central ideas using the Collective Subject Discourse (CSD) method [43]. First, each researcher conducted their own data analysis, selecting the terms that, in their view, best represented the central themes. Subsequently, both researchers met to compare and discuss the selected terminology, evaluating each term to decide which best represented the respondent ideas. During these discussions, a 90.0% agreement rate was achieved between the analyses. In cases of divergence, the reasons behind each term's proposal were thoroughly examined. The researchers then consulted an online Portuguese dictionary and, afterwards, an online English dictionary [44] to ensure translation accuracy and the semantic precision of the terms. Finally, these terms were incorporated into the word clouds, where the most frequent terms appear in proportionally larger sizes” (lines 207-217).

Reviewer 2, we sincerely appreciate the thorough evaluation and valuable suggestions, which were essential for improving this work. Each comment and recommendation was carefully considered, and the changes implemented aim not only to address the observations made but also to enhance the clarity and depth of the research. We are grateful for the time dedicated to the review and for the constructive and enriching feedback provided.

Please do not hesitate to contact us if any further information is required.

Sincerely,

The Authors.
