# Supplementary material for: How much do opinions regarding cultivated meat vary within the same country? The cases of São Paulo and Salvador, Brazil
Source: PLoS One. 2025 Feb 19;20(2):e0317956. doi: 10.1371/journal.pone.0317956 (PMC11838885; doi:10.1371/journal.pone.0317956)
Supplement: Attachment — Submitted filename: Response_to_Reviewers_auresp_2.docx [file pone.0317956.s004.docx]

Curitiba, December 18^th^, 2024

Dear Editor,

We sincerely appreciate your consideration of our manuscript and the constructive feedback provided by the reviewers. We are pleased to know that the work has merit, and we thank you for the invitation to submit a revised version addressing the points raised during the review process. We have carefully considered all recommendations and have addressed each one; we believe that the revisions resulted in a significantly improved manuscript.

We confirm that no changes were made to our financial disclosure statement. We inform you that the reference list has been reviewed to ensure it is complete and correct.

Dear Reviewers,

We sincerely thank you for your detailed evaluation and thoughtful feedback. Your valuable suggestions have greatly contributed to enhancing the quality and clarity of the manuscript. Each recommendation has been carefully reviewed and addressed, and we are confident that the revisions have substantially improved the manuscript.

For clarity, the reviewers' comments are highlighted in *italic*.

Reviewer 1:

*In the introduction section, I appreciate the extensive references to the literature. However, I believe the introductory section should be kept distinct from the theoretical background section. That said, while the authors have included an overview of the paper's structure at the end of the introductory paragraph, it is not very clear. Therefore, I suggest that the authors rewrite this part to make it more concise and clearer.*

We have rewritten the overview of the paper's structure at the end of the introductory paragraph, making it more concise and clearer: “The paper begins by providing a demographic profile of the participants, followed by a detailed presentation of descriptive and qualitative data derived from their responses. These data are then summarized and analyzed using quantitative methods, including binary logistic regression and ordinal regression models, as well as the qualitative Collective Subject Discourse (CSD) [43] methodology. The analysis highlights comparisons between the two cities while considering demographic variables as potential explanatory factors. Finally, the discussion section addresses the study's findings, moving from general observations to more specific insights. It also outlines the study's limitations and suggests priorities for future research in the field” (lines 160-168).

*The description of the methodology is clear in most parts, but some points can be enhanced for better understanding:*

*The timing of the survey (September 5th to 23rd, 2019) is clearly stated, but it could be useful to briefly mention how the participants were selected within the cities, for example, whether they were chosen randomly, or if there were any stratifications or quotas used based on demographics.*

We have now clarified that the participants were recruited randomly within the cities (line 173).

*You mention considering various factors (city, income, education, and frequency of meat consumption) as explanatory variables. It would be beneficial to explain why these particular variables were selected. For example, what prior research or reasoning suggests that these variables are most likely to influence consumer attitudes toward cultivated meat?*

We have expanded the discussion to explain the selection of these variables: “selected for their possible relationship with conventional meat consumption and the intention to consume cultivated meat, according to previous studies [13, 22, 29, 31]” (lines 185-187).

*The statistical methods seem appropriate, but I would recommend providing a brief explanation or justification for the use of the "odds ratios" and "proportional odds regression models." For readers unfamiliar with these methods, a short explanation on why these specific models were chosen could be beneficial.*

We have added a brief explanation to justify the use of odds ratios and proportional odds regression models. This addition clarifies that these methods were chosen for their ability to analyze ordinal outcomes and quantify the relationship between explanatory variables and the likelihood of different levels of consumer attitudes, making them particularly suitable for our study: “The statistical methods used, such as odds ratios and proportional odds regression models, are appropriate for analyzing the relationship between independent variables and the likelihood of categorical events, like the intention to consume cultivated meat. Odds ratios measure the effect of explanatory variables on event probabilities between two groups, while proportional odds regression models are useful for comparing outcomes with more than two ordered categories. These models were chosen for their ability to handle categorical data and proportional responses, common in consumption and attitude studies” (lines 225-231).

*In my opinion, the revised version of the Discussion section is clearer and more concise. Despite that, while the key findings are highlighted, the connection between these findings and the broader context could be better clarified. In particular, explaining why respondents from Salvador and São Paulo differ in their knowledge and acceptance of cultivated meat could be strengthened by referencing social, economic, and cultural factors. For instance, you could explain that Salvador, with its higher unemployment rate and lower income levels, may have less access to information about new technologies, influencing their awareness and attitudes toward cultivated meat.*

To improve the connection between the key findings and the broader context, we addressed the differences between respondents from Salvador and São Paulo, referring to social, economic, and cultural factors: “Salvador, with its higher unemployment rate and lower income levels, may have more limited access to information about new technologies, which in turn may influence the population's awareness and attitudes toward cultivated meat” (lines 359-361); “Residents of São Paulo live in a city with a more diversified economy and greater access to technologies and innovations, which likely offers more opportunities for being constantly informed about new food alternatives, such as cultivated meat. Additionally, the higher purchasing power and greater environmental awareness in São Paulo may positively influence the acceptance of alternative products, like cultivated meat. In contrast, citizens of Salvador, facing economic challenges such as a higher unemployment rate and lower purchasing power, may encounter barriers to accessing information about these new products, which could result in a lower intention to consume” (lines 374-382).

*Furthermore, I think that smoother transitions would improve the flow. For example, after discussing regional knowledge gaps, you could smoothly transition into the sensory properties of cultivated meat by noting how sensory factors, like taste and texture, play a critical role in consumer acceptance once familiarity with the product increases. You might use transitional phrases like "In addition to regional differences in awareness, sensory perceptions also shape acceptance," which would help link these ideas more cohesively.*

We revised the Discussion section to improve the flow by adding smoother transitions between ideas: “This trend is reflected in the intention to consume cultivated meat, which was lower” (line 365); “In addition to regional differences in awareness, sensory perceptions also influence the acceptance of cultivated meat. Apparently, people are motivated to try cultivated meat mainly out of curiosity regarding its organoleptic properties, such as taste and texture” (lines 384-386); “Moving from sensory perceptions to broader motivations, animal welfare concerns also play a significant role in the growing interest in consuming cultivated meat” (lines 397-398); “Alongside animal welfare, human health frequently drives the desire for cultivated meat. However, many respondents, particularly those unfamiliar with cultivated meat, believe conventional meat to be more health beneficial” (lines 409-411); “In terms of consumer demographics” (line 448); “While younger, wealthier, and more educated consumers tend to show more positive views towards cultivated meat, there are significant barriers to widespread acceptance, such as food neophobia” (lines 463-465); “As consumers become more familiar with and educated about cultivated meat, the transition from conventional to alternative protein sources may become smoother. This shift, however, is not without challenges” (lines 489-491).

Overall, we deeply appreciate the detailed evaluation, which was crucial in improving the work. We considered all the suggestions and made the necessary adjustments to strengthen the analysis and clarity of the results. All comments were carefully reviewed and incorporated to ensure the work met the high standards of excellence required by PLoS One.

Reviewer 2:

*I recommend including a concise description of the methods used to process and analyze data, as this information is currently missing from both abstract and introduction. For instance, quantitative methods such as binary logistic regression or ordinal regression models, and qualitative methods like the collective subject discourse methodology (CSD), could be briefly mentioned. The revised abstract is lengthy, I recommend balancing conciseness with essential information.*

We made the necessary adjustments, including a concise description of the methods used to process and analyze the data. In the abstract, we briefly mention the quantitative methods, such as binary logistic regression and ordinal regression models, as well as the qualitative method, the collective subject discourse methodology (CSD). We also adjusted the distribution of sections in the abstract, ensuring that all parts of the study were represented clearly and in balance.

“An online questionnaire comprising 17 multiple-choice and open-ended questions about opinions on conventional and cultivated meat was administered. The results were analyzed using quantitative methods, including binary logistic regression and ordinal regression models, as well as the qualitative Collective Subject Discourse methodology” (lines 31-34).

*In the introduction, the methods are only mentioned in the final paragraph as 'appropriate methodology for each case.' Adding a brief and clear description of the methods in this section would provide readers with a better understanding of the analytical approaches used in the study.*

We revised the introduction to include a brief and clear description of the methods used in the study: “The paper begins by providing a demographic profile of the participants, followed by a detailed presentation of descriptive and qualitative data derived from their responses. These data are then summarized and analyzed using quantitative methods, including binary logistic regression and ordinal regression models, as well as the qualitative Collective Subject Discourse (CSD) [43] methodology. The analysis highlights comparisons between the two cities while considering demographic variables as potential explanatory factors” (lines 160 -165).

*The inclusion of tables significantly enhances the readability of the results. However, it would be beneficial to present each table either before or after a brief paragraph that summarizes the main findings, ensuring cohesion and flow within the text.*

Thank you for the suggestion. We have implemented it in the first round of revisions by presenting the results in table format to provide greater clarity and facilitate interpretation. The key results from each table can be found in the paragraphs starting at lines 249, 260, 270, and 292, as well as in figures 1 and 2.

*The paragraph included at the end of the discussion section effectively highlights the study's limitations, particularly those stemming from the challenges of researching public opinions on topics that are not widely known by the population.*

*The inclusion of Table 5, 'Responses to questions about the relationship between cultivated meat and the environment, human health, and animal welfare, based on respondents' familiarity with cultivated meat,' is valuable for identifying differences within the sample between respondents who answered YES and those who answered NO. However, this table requires a more in-depth interpretation or further mention in the discussion section to demonstrate its contribution to the study’s findings.*

We made the necessary adjustments and included a more in-depth interpretation of Table 5 in the discussion section, highlighting its contribution to the study’s findings and the relevance of the differences between respondents who answered “yes” and those who answered “no”: “Whether or not they were familiar with cultivated meat, respondents generally believed that it is more beneficial than harmful to animal welfare” (lines 399-401); “People perceive that the introduction of cultivated meat to the market may reduce, or at least prevent the worsening of, animal suffering caused by factory farming, as this alternative meat production system does not require the slaughter of animals [22, 31]” (lines 404-407); “However, many respondents, particularly those unfamiliar with cultivated meat, believe conventional meat to be more health beneficial” (lines 410-411); “Prior knowledge of the similarities between cultivated and conventional meat, differing mainly in their production methods, may help clarify the perception that cultivated meat is more harmful to human health than conventional meat” (lines 421-424); “Even among those who were not familiar with cultivated meat, many considered it more beneficial than harmful to the environment” (lines 431-432); “as even those who were not yet familiar with it perceived it positively in terms of its environmental impact” (lines 442-443);

*The authors provide additional details about the methodology used to generate the word clouds. Since the suggestion to exclude certain custom words was not addressed, I recommend including a brief explanation to highlight the scope of the word cloud analysis.*

We included a brief explanation in the methodological section to highlight the scope of the word cloud analysis. Additionally, we clarified the criteria used in the selection of the words: “The selection of words for the cloud was based on two main criteria: their technical meaning and relevance in the context of the research, as well as their general meaning according to the dictionary” (lines 216-219); “For conventional meat, the main ideas in response to question 8 cited by the 809 respondents were i) production (21.6%), with words like farm, production, and reproduction; the word “production” was chosen because it encompasses the raising of animals for food, including reproduction and the process of breeding species, which is central to the meat industry; ii) suffering (11.7%), with citations such as cruelty, torture, suffering; the word "suffering" was chosen because it encompasses the terms mentioned by respondents when expressing their opinions on the treatment of animals in the conventional production system; iii) logistics (11.6%), which includes words such as distribution, transport, logistics; the word "logistics" reflects the process related to the logistical aspects of the conventional meat production supply chain. For cultivated meat, the main answers given involved i) innovation (12.1%), with words like technology, advance, innovative; the word "innovation" was highlighted due to its association with technological advances and the idea of something new and progressive mentioned by the respondents; ii) neophobia (10.8%), with words such as strange, fear, distrust; the word "neophobia" was chosen to represent the resistance and fear of new technologies expressed by the respondents; iii) artificial (8.2%), containing words such as false, unnatural, artificial; the word "artificial" was chosen as it is synonymous with the ideas of artificiality and unnaturalness expressed by the respondents” (lines 313-329).

Overall, we deeply appreciate the careful evaluation and valuable suggestions, which were essential for improving this work. Each comment and recommendation were thoughtfully considered, and the changes made aim not only to address the observations but also to enhance the clarity and depth of the research. We are grateful for the time dedicated to the review and for the constructive and enriching feedback provided.

Please do not hesitate to contact us if any further information is required.

Sincerely,

The Authors.
