# Supplementary material for: How much do opinions regarding cultivated meat vary within the same country? The cases of São Paulo and Salvador, Brazil
Source: PLoS One. 2025 Feb 19;20(2):e0317956. doi: 10.1371/journal.pone.0317956 (PMC11838885; doi:10.1371/journal.pone.0317956)
Supplement: Attachment — Submitted filename: Response_to_Reviewers_auresp_3.docx [file pone.0317956.s005.docx]

Curitiba, January 02^nd^, 2025

Dear Editor,

We would like to express our sincere gratitude for your consideration of our manuscript and the constructive feedback provided by the reviewers. We are pleased that the work has been well-received, and we appreciate the invitation to submit a revised version that addresses the points raised during the review process. We have thoroughly reviewed all the recommendations and made the necessary revisions, which we believe have substantially improved the manuscript.

We confirm that no changes have been made to our financial disclosure statement. Additionally, we have reviewed the reference list to ensure its accuracy and completeness.

Dear Reviewers,

We deeply appreciate your thorough evaluation and insightful feedback. Your invaluable suggestions have significantly contributed to improving both the quality and clarity of the manuscript. Each of your recommendations has been carefully considered and incorporated, and we are confident that the revisions have greatly enhanced the manuscript.

For clarity, the reviewers' comments are highlighted in *italic*.

Reviewer 1:

*The authors have thoroughly addressed all comments and suggestions provided during the review process. In my opinion, there is no further need for improvement. As the paper also meets the publication criteria of PLOS ONE, I consider it accepted for publication.*

Dear reviewer 1, we would like to express our sincere and profound gratitude for the valuable suggestions and comments provided. Your detailed and relevant observations undoubtedly played a key role in the substantial improvement of the work, allowing us to enhance several important aspects of the research. We greatly appreciate the time and effort dedicated to reviewing the manuscript, and we are confident that the modifications made in response to your recommendations have significantly strengthened the quality and clarity of the content presented.

Reviewer 2:

*Although the authors have added tables in the initial revisions, the latest suggestion to enhance the description of the results presented in these tables has not been considered. I recommend identifying key insights (including those related to demographics) and comparing data between cities, given the paper's emphasis on the variability of opinions within the same country. This analysis is critical and needs careful articulation to ensure clarity, smooth transitions, and emphasis on key findings. Additionally, the titles of tables and figures contain unnecessary details such as “809 respondents in an online questionnaire from September 5 to 23, 2019”. Such specifics should be presented in the materials and methods section.*

We adapted the demographic data table (Table 2) to compare information between cities. Additionally, we included a brief paragraph before each table, highlighting the main findings: “When respondents were asked if they had heard about cultivated meat, 310 out of 809 participants (38.3%) answered yes (Fig. 1). In São Paulo, 187 respondents (44.6%) reported familiarity with cultivated meat, compared to 123 (31.5%) in Salvador. Table 3 presents the results of this question broken down by demographic variables” (lines 258-261); “Overall, 265 participants (32.8%) expressed interest in eating cultivated meat, 211 (26.0%) indicated they would not consume it, and 333 (41.2%) responded "I don't know" (Fig. 1). In São Paulo, 170 respondents (40.6%) said they would eat cultivated meat, compared to 95 respondents (24.4%) in Salvador. Table 4 presents the results of this question, categorized by demographic variables” (lines 268-272); “Among respondents who were already familiar with cultivated meat, 205 (66.1%) believe it benefits animal welfare, 108 (34.8%) consider it beneficial for human health, and 174 (56.1%) for the environment. In contrast, 215 (43.1%) among those who had never heard of cultivated meat, believe it benefits animal welfare, 75 (15.0%) see benefits for human health, and 199 (39.9%) for the environment (Table 5)” (lines 294-298).

As for the details provided in the title of tables, we agree that this information has already been presented in the Material and Methods section. However, we decided to maintain it because we understand that it is important that readers get the full information when looking at tables or figures, independently from reading the text. We also want to consider the opinion of the reviewer who has already accepted the paper as it is, by avoiding the deletion of any part of the manuscript.

*The authors have addressed this suggestion by identifying relevant differences and similarities between both groups in the discussion section. However, as this point relates to the previous suggestion, I recommend first clarifying these key insights in the results section. They should then be further analyzed within the discussion section to enhance the understanding of their implications in the broader context.*

We clarified the main findings from Table 5 in the Results section, structuring them according to the sequence in which they are discussed throughout the text. In the Discussion section, we address the results related to animal welfare, human health, and the environment, between lines 406 and 454.

*The authors have provided valuable clarifications about the technical criteria, which enhance the understanding of the process used to generate the word clouds. However, the contribution of these word clouds to the overall study remains unclear, as there is no analysis of them in the results section. Although their primary function—to identify the most frequent words—might seem obvious, explicitly stating and discussing this purpose within the results would clarify their role in the study.*

We explained the purpose and role of Figure 3, which presents the word clouds, in the Results section, highlighting its contribution to the data analysis: “The respondents were asked to share the three words that came to their minds when thinking about the production systems of conventional and cultivated meat. Given the large volume of data, after analyzing each term, a word cloud was generated with the words that best represented the ideas expressed by the participants. For conventional meat, the words that best reflected the ideas mentioned by the participants were…” (lines 316-320); “For cultivated meat, the words that best reflected the ideas mentioned by the participants were…” (lines 328-329).

In summary, we sincerely appreciate the meticulous evaluation and insightful suggestions, which have been instrumental in enhancing this work. Each comment and recommendation was carefully reviewed, and the revisions implemented are intended not only to address the observations but also to strengthen the clarity and depth of the research. We are grateful for the time and effort invested in the review process, as well as for the constructive and enriching feedback provided.

Please do not hesitate to contact us if any further information is required.

Sincerely,

The Authors.
